# MENTAL HEALTH AS A FORTITUDE FACTOR

**DOI:** 10.1093/geroni/igae098.3436

**Published:** 2024-12-31

**Authors:** Tobi Abramson, Jackie Berman, Karen DeBell, Christina Hatten

**Affiliations:** NYC Department for the Aging, New York, New York, United States; NYC Department for the Aging, New York, New York, United States; New York State Office of Mental Health, Albany, New York, United States; New York State Office of Mental Health, Albany, New York, United States

## Abstract

NYS’ Geriatric Mental Health Act has led to the establishment of a series of Geriatric Demonstration grants. To build fortitude, this round of grants focuses on the development of a Partnership to Support Aging in Place (PSAP) as a model to provide in-community services and supports to address unmet behavioral health, physical health, aging, and social support needs of older adults. The triple partnership of providers includes three core service areas: (1) OMH licensed mental health services (2) OASAS licensed SUD service providers (3) NYS Area Agency on Aging (AAA). The grant components also includes providing certified peer specialists, utilizing technology, and wrap around funding. These grants address disparities in access to services experienced by diverse, vulnerable, marginalized populations, those disproportionately affected by the COVID-19 pandemic. For the 6 grants, there have been over 280 admissions of older adults who need either mental health, aging services, or substance use disorder services. Of enrollees, 72% suffered from depression, 73% suffered from anxiety, 27% suffered from substance abuse, and 50% had an aging need. Innovative technologies includes the implementation of a created curriculum surrounding Fitbit use, and Elli-Q. Both substance use disorder and mental health peers have been central in these grants and many of these peers have been instrumental in conducting the peer-led program known as ‘Do More Feel Better’. Each component of this initiative and the outcome data will be discussed in this session. All of these factors strengthen older adults mental health fortitude.

